# Assessment of the impact of the malaria elimination programme on the burden of disease morbidity in endemic areas of Iran

**DOI:** 10.1186/s12936-016-1267-9

**Published:** 2016-04-14

**Authors:** Khodadad Sheikhzadeh, Ali Akbar Haghdoost, Abbas Bahrampour, Farzaneh Zolala, Ahmad Raeisi

**Affiliations:** Research Center for Modeling in Health, Institute for Futures Studies in Health, Kerman University of Medical Sciences, Kerman, Iran; Regional Knowledge Hub, and WHO Collaborating Center for HIV Surveillance, Institute for Futures Studies in Health, Kerman University of Medical Sciences, Kerman, Iran; Department of Medical Entomology and Vector Control, School of Public Health, Tehran University of Medical Sciences, Tehran, Iran

**Keywords:** Malaria elimination, Iran, Poisson, Multilevel

## Abstract

**Background:**

Controlling and preventive measures considerably reduced malaria incidence in Iran over the past few years, which confined the endemic areas to some regions in the southeastern Iran. The National Malaria Elimination Programme commenced in 2010. With regard to the presumption that the elimination programme interventions have accelerated the declining trend of malaria incidence across the endemic areas of Iran, the present study attempted to assess the effectiveness of the elimination programme by reviewing malaria incidence status, over a 14-year period, and comparing the trend of malaria incidence across malaria-endemic areas between the control and pre-elimination phase, and the elimination phase.

**Methods:**

A retrospective analysis of malaria surveillance data was conducted in a 14-year period (2001–2014), using multilevel Poisson regression. The epidemiological malaria maps and indicators also were developed and compared between the control and pre-elimination phase, and the elimination phase.

**Results:**

The mean of malaria incidence was 2.2 (1.7–2.7) for the entire study period. This rate was 3.4 (2.6–4.1) in the control and pre-elimination phase, and 0.41 (0.25–0.57) for the elimination phase. During the malaria elimination phase, the decline of annual malaria incidence had significantly accelerated and autochthonous cases had the greatest difference in malaria incidence decline (compared to the control and pre-elimination phase), whereas, falciparum cases had the lowest difference in malaria incidence decline, followed by non-Iranian and imported cases. Furthermore, there was a decline in Iranians to non-Iranians ratio and an increase in the ratios of over 15 to under 15, as well as male to female, in the elimination phase in comparison to the control and pre-elimination phase.

**Conclusions:**

It seems that the decline of malaria transmission, which has been initiated over the past few years, has accelerated as a result of the elimination programme, and Iran is approaching the goals set regarding the elimination of this disease.

## Background

Despite all the efforts worldwide, malaria is still going strong as one of the world’s health challenges, especially in developing countries, such that it was set as one of the Millennium Development Goals [1]. The latest estimation by World Health Organization reported 214 million cases of malaria and 438,000 deaths in 2015 [2]. Despite the extent and severity of the problem, a review of the trends of malaria cases during the past years indicates desirable progress in reducing the incidence and death rates.

Although African countries bear the major burden of malaria, it has long been a health issue in many other countries, including Iran. Prior to the implementation of control programmes, more than 60 % of the Iranian population lived in malaria-endemic areas, and four to five million cases of malaria were reported annually [3]. The highest incidence in the past 40 years occurred in 1991, when 98,160 cases of malaria were reported across the country.

In recent years, the number of cases of malaria in Iran has declined. In 2014, only 1230 cases were, of which about 22 % were of local transmission. Cases of falciparum malaria only represented 9 % of the total cases. A considerable portion of the annually reported, as well as locally-transmitted, cases in the past few years originate from counties located in three south-eastern provinces.

The first feasibility study of malaria elimination in Iran was conducted in 2007. The National Malaria Elimination Programme was developed by 2008, and it was finally approved and officially commenced in 2010. Phase one of the programme, as a 5-year long strategic plan, was implemented from 2010 to 2014, and phase two was planned for 2015–2019. In the control phase, according to Roll Back Malaria, the main interventions were: using vector control activities (such as distribution of insecticide-impregnated bed nets, larviciding and indoor residual spraying), early diagnosis and prompt treatment, epidemiologic classification of malaria cases, community education, early detection and control of malaria epidemics, using county stratification in order to select proper interventions in each stratum. In the elimination phase, however, all the above activities are intensified. In addition to that, other measures are carried out in the elimination phase which includes: epidemiologic classification of all the foci and malaria cases, intensified and targeted vector control activities, political advocacy, vigilance case notification, targeted distribution of long-lasting insecticide-impregnated bed nets, extended use of rapid diagnostic tests, involvement of community volunteers, establishment of emergency sites (with enough necessary equipment and materials such as thermal fogs, sprayers, insecticide, etc.) and rapid response teams in endemic areas in order to rapid response to malaria outbreaks [4, 5].

Elimination of local transmission of falciparum malaria by 2018, and vivax malaria by 2022, and ultimately, attaining the goal of malaria elimination across the country and being awarded Certification of Elimination by 2025, are all goals of phase two of the programme [6].

With regard to the fact that the interventions of the elimination programme have accelerated the declining trend of malaria incidence, as well as, local malaria transmission across endemic areas of Iran, assessment of effectiveness of the elimination programme and the related interventions on the accelerated decline of cases across malaria-endemic areas gains significance. Accordingly, comment on the interventions and their overall impact in fulfilling the designated goals of the elimination programme, could be given based on such an assessment.

The retrospective study on malaria incidence, aiming at determining the status and planning for malaria elimination, has been conducted across many regions [7, 8]. A number of studies have also been conducted across malaria-endemic areas in Iran, indicating a reduction in the incidence in the past recent years [9]. The majority of these studies, however, have addressed small areas. Review of the related literature did not reveal any studies in which the pre-elimination and elimination periods have been compared.

The present study assessed the effectiveness of the elimination programme, by reviewing malaria incidence status in a 14-year period, and comparing cases across malaria-endemic areas in the control and pre-elimination phase (CP) as well as the elimination phase (EP).

## Methods

### Area of the study

**Fig. 1:**
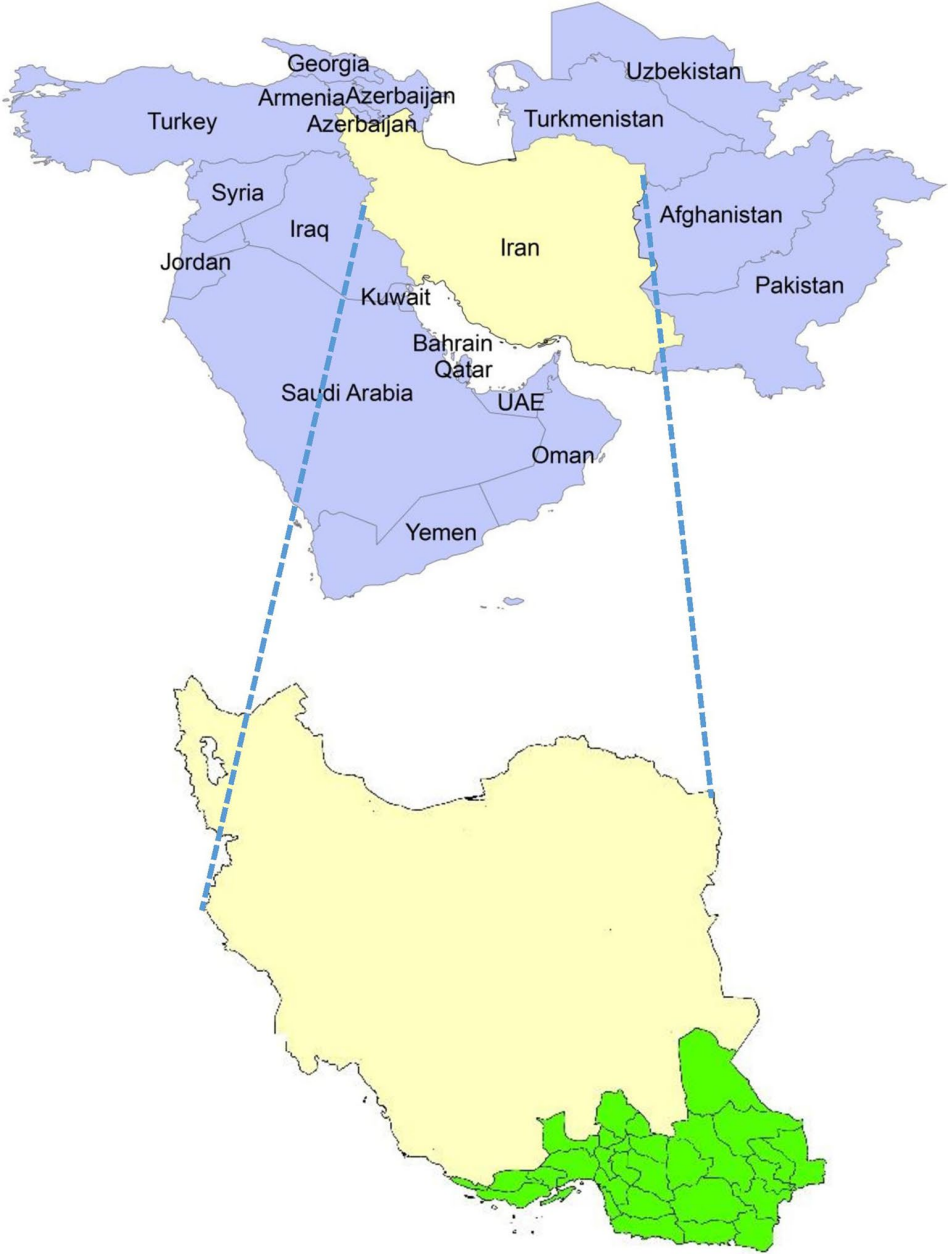
Area of the study

Area of the present study is south-eastern Iran, including 30 counties in three south-eastern provinces, across an area of 271,878 km^2^ (16.4 % of Iran’s land area) (Fig. 1). Total population of this area was slightly over 3,500,000 individuals (4.6 % of Iran’s total population).^1^ This area borders on Pakistan towards east, connects to the Persian Gulf and Oman Sea to the south, and neighbors the non-endemic inland areas towards the west and north. These so-called endemic areas have a potential for transmission of malaria due to high-traffic border and population displacement across with the eastern neighbouring countries, suitable climatic conditions for malaria vectors survival, and low socioeconomic conditions. In other areas across the country, the reported cases are, mainly, imported cases from the endemic areas or non-Iranians. This area suffers from unstable malaria with *Plasmodium vivax* (the dominant parasite) and *Plasmodium falciparum*.

### Data collection and management

A retrospective analysis was conducted on malaria surveillance data. To do this, data from the malaria surveillance system were collected from all the health centres across the areas of the study during a 14-year period (2001–2014). All the recorded cases of malaria in Iran were initially approved by microscopic examination and then recorded at health centres. Accordingly, the incidence data and pertaining demographic indicators (namely, age group, sex, nationality, manner of detection), were collected from all the health centres in the area of study for each and every year divided by the parasite (*P.vivax* and *P.falciparum*) and modes of transmission (imported versus autochthonous).^2^ These data were extracted and collected from malaria surveillance programme forms recorded at the health centres of malaria-endemic areas. Two trained and experienced health staff collected data to ensure quality of data gathering and recording. Also, the data were simultaneously monitored and examined through cross-referencing to the data bank of the Center for Disease Control at the Ministry of Health, as well as consulting with the resident medical experts. Any mismatches were reported for correction. Subsequent to data collection, for county-scale analysis, data from the health centres of the studied counties were integrated. In an attempt to prepare the incidence maps for the counties, considering the new established counties during the study period, the existing shape files were edited to match the latest geographical breakdown. Maps for the geographical distribution of the disease were prepared using identical scales to enable comparison over time and across different areas. Annual population changes of the counties during the period of study were extracted for the calculation of incidence.

### Data analysis

Considering the fact that the data were numerical and followed a Poisson distribution, simple and multilevel Poisson models were compared, and due to significant difference, multilevel Poisson regression was adopted for data analysis. Since malaria elimination programme commenced in 2010, a variable was introduced to differentiate the time before the implementation of elimination programme, which routine control activities were put into the practice (control and pre-elimination phase-CP) and the elimination phase (EP), in an attempt to study and compare the trends of malaria incidence during the mentioned two phases. Decline of malaria incidence was also compared in the two phases to reveal whether or not these reductions were significant, or, otherwise put, to determine whether the malaria elimination programme has managed to accelerate the incidence decline.

Furthermore, according to the collected data, the epidemiological indicators were developed and compared, divided by each time phase. Confidence interval was calculated for all indicators.

Arc GIS 9.3 was used to develop incidence maps for 2009 (1 year before commencing the programme) and 2014, for later comparison. Stata 11 was adopted for data analysis.

### Ethical considerations

None of the collected data bore any names or personal particulars; nonetheless, the required permits were obtained from the Ministry of Health, and Universities of Medical Sciences in the areas of the study.

## Results

**Fig. 2:**
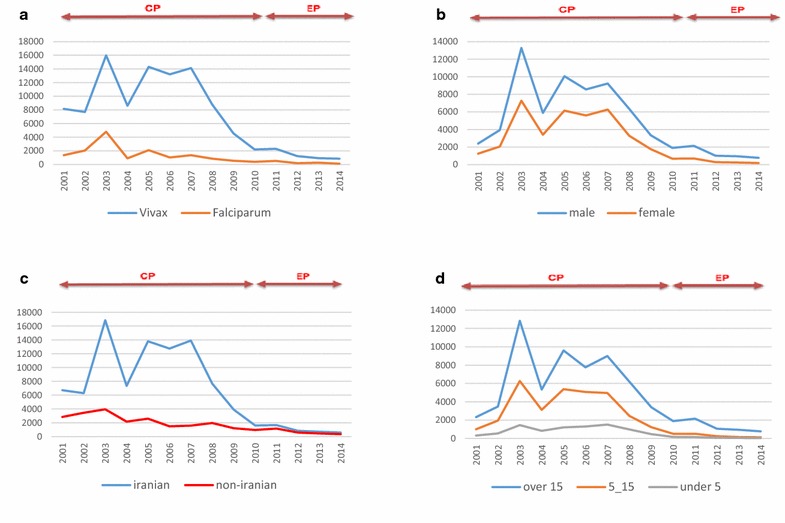
Trends in malaria cases in the endemic areas of Iran by parasite species (**a**), gender (**b**), nationality (**c**), age group (**d**) and 2001–2014

The present study was conducted on 119331 positive cases of malaria, within a 14-year period, across two time intervals: CP (2001–2009) and EP (2010–2014). The time trends of malaria incidence indicate a decline in the incidence of vivax and falciparum malaria during the study period (Fig. 2).

**Fig. 3:**
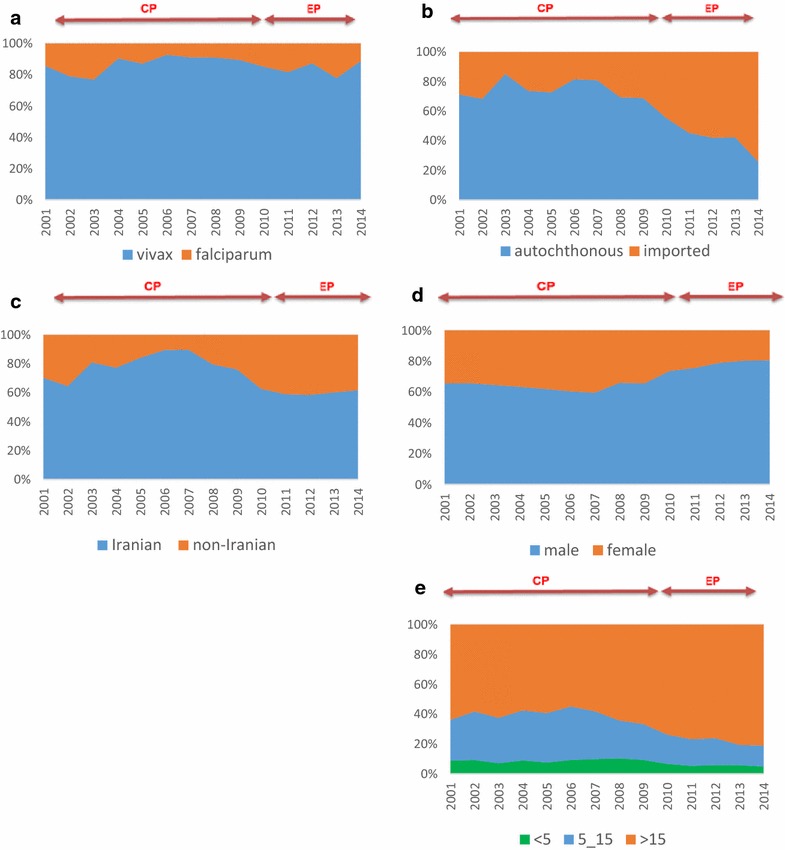
Malaria indices (%) in the endemic parts of Iran according to parasite species (**a**), transmission routes (**b**), nationality (**c**), sex (**d**) and age (**e**); (2001–2014)

During the study period, percentage of male cases, cases over 15, and imported cases gradually increased (Fig. 3). The mean of malaria incidence (per thousand population) was 2.2 (1.7–2.7) for the entire study period. The same rate was 3.4 (2.6–4.1) in the CP and 0.41 (0.25–0.57) for the EP. Males formed 64 % (62–66 %) of the positive cases, and 61 % (59–64 %) of the cases were over 15. The corresponding rates were 63 % (61–65 %) and 60 (57−62 %) for the CP, and 76 % (73−78 %) and 77 % (75–78 %) for the EP, respectively. The majority of the cases were caused by autochthonous transmission (75 %). Autochthonous transmission cases were 77 % (72–81 %) for the CP and 45 % (40–51 %) for the EP. An approximate overall of 79 % of the cases were Iranian citizens, respectively, at 81 % (77–85 %) and 61 % (56–65 %) for the CP and EP. *P. vivax* was the most prevalent (86 %) species.

Overall malaria incidence (regardless of the time trend) showed an 88 % decline in the EP, compared to the CP. Since the declining trend of incidence had started prior to the interventions, a complementary analysis was conducted to examine the decline trend in the CP and EP, the results of which showed that prior to the commencement of malaria elimination programme, malaria-endemic areas showed an 8 % annual malaria incidence decline, on average (IRR^3^ = 0.919, Cl 0.917–0.922). Annual decline of overall malaria incidence was increased in the EP (26 % per year), which was significant (*P* value = 0.0001). Investigation of the incidence status and annual decline ratio of vivax malaria cases, shows a more or less similar pattern, indicating a significant difference between the annual decline rates between these two time periods (Table 1).

**Table 1:** The impact of elimination programme on malaria incidence in Iran; comparison of two time periods

	Pre-elimination phase^a^				Elimination phase^a^				Interaction^c^ (*P* value)
	IRR^b^	CI		*P* value	IRR	CI		*P* value	
Total cases	0.919	0.917	0.922	0.0001	0.738	0.727	0.750	0.0001	0.0001
*P. vivax*	0.938	0.936	0.940	0.0001	0.739	0.727	0.751	0.0001	0.0001
*P. falciparum*	0.800	0.795	0.806	0.0001	0.735	0.708	0.764	0.0001	0.0001
Autochthonous^d^	0.924	0.922	0.927	0.0001	0.645	0.630	0.661	0.0001	0.0001
Imported	0.907	0.903	0.912	0.0001	0.817	0.800	0.833	0.0001	0.0001
Iranian	0.943	0.940	0.945	0.0001	0.734	0.719	0.749	0.0001	0.0001
Non-Iranian	0.823	0.818	0.827	0.0001	0.744	0.726	0.762	0.0001	0.0001

^a^Pre-elimination phase: 2001–2009; Elimination phase: 2010–2015

^b^Incidence Rate Ratio for the linear effects of time (year), which indicates that how much malaria incidence of a certain year has changed compared to the previous year, on average. A value of 1 indicates that the incidence has remained roughly constant, and values less than 1 indicate a declining trend in the incidence; the more this value grows smaller, the higher will be the decline rate

^c^Interaction between phase and year; the small P-value (less than 0.05) of this coefficient indicates the significant difference of the risk ratio of annual incidence decline in the pre-elimination to the elimination phase, it can, therefore, be concluded that for all the compared groups, the speed of annual decline rate in the elimination phase was significantly higher than the previous phase

^d^Local malaria transmission in the endemic counties

The decline in the incidence of falciparum malaria was faster than that of *vivax* malaria in the CP (an annual decline of around 20 %), which was accelerated in the EP, to simulate the decline of incidence of *vivax* malaria. The difference between the decline rates of falciparum malaria in the two phases was also significant. Monitoring autochthonous transmission incidence reveals that though annual decline in autochthonous transmission cases roughly matched total malaria cases in the CP, it possessed the greatest decline rate in the annual cases, subsequent to commencing the elimination programme (IRR 0.645, CI 0.630–0.661). The imported cases show a lower decline in the EP (Table 1).

**Fig. 4:**
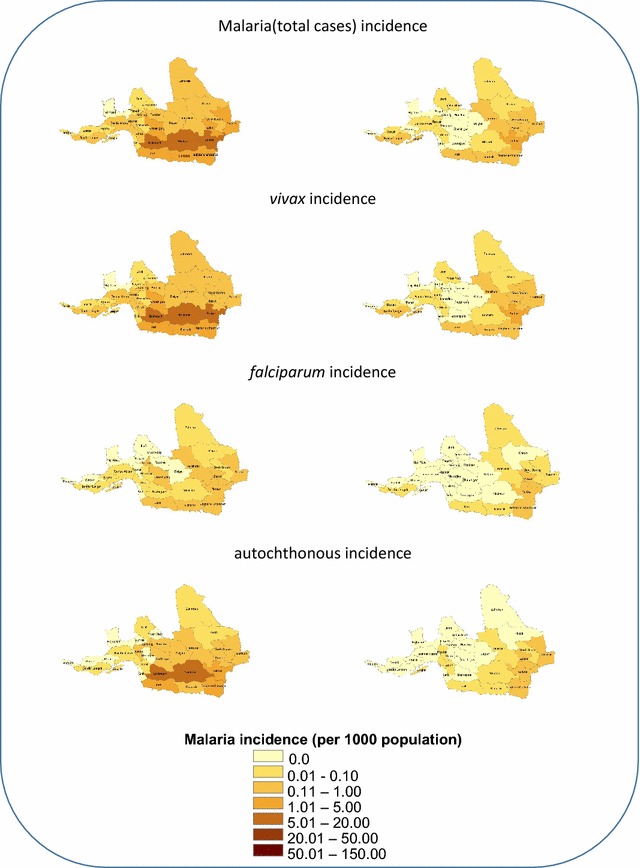
Comparison of geographical distribution of annual malaria incidence between 2009, before starting the elimination phase (the *left column*), and 2014 (the *right column*)

A comparison of the geographical distribution of cases across malaria-endemic areas in 2009 (the year before commencement of elimination programme) and 2014 (last year of the study period) shows that during the EP, the central and western counties of the study area have approached the elimination target, wherein incidence is either zero or minimal. Whereas, eastern and border areas still suffer from a higher incidence rate, compared to other areas (Fig. 4). The ratio of Iranian to non-Iranian cases in the EP compared to CP shows a decline for vivax and falciparum cases, as well as the total cases (Table 2). Comparing positive male to female cases indicates an increase in this ratio in the EP. Also, the ratio of cases over 15 to cases under 15 in the two periods shows similar results, which indicates an increase in percentage of over 15 age group, compared to the under 15 group, in the EP. Examining the manner of detection of malaria cases in the two periods revealed a decline in the case detection ratio (active to passive) for *vivax* cases in the EP; yet, an increase in the mentioned ratio for falciparum cases (Table 2).

**Table 2:** Comparison of different malaria indices (CI) between control and pre-elimination phase (CP) and elimination phase (EP) in endemic areas of Iran, by parasites species and transmission routes—2001–2014

Parasite	Transmission	Nationality Iranian/non-Iranian ratio^a^		Sex Male/female ratio^a^		Age over 15/under 15 ratio^a^		Case detection^b^ Active/passive ratio^a^	
		CP	EP	CP	EP	CP	EP	CP	EP
*Vivax*	A	16.8 (8.7–24.9)	4.9 (2.9–6.8)	1.5 (1.4–1.6)	2.3 (1.9–2.6)	1.3 (1.1–1.4)	2.5 (2.0–2.8)	1.5 (1.2–1.7)	1.1 (0.9–1.3)
	I	1.0 (0.8–1.1)	0.8 (0.6–0.9)	2.8 (2.4–3.2)	4.4 (3.9–4.9)	2.3 (1.8–2.8)	4.0 (3.5–4.5)	0.5 (0.4–0.5)	0.7 (0.6–0.8)
	S	5.9 (4.2–7.6)	1.7 (1.2–2.0)	1.6 (1.5–1.8)	3.1 (2.7–3.5)	1.4 (1.3–1.6)	3.1 (2.8–3.5)	1.2 (1.0–1.4)	0.9 (0.8–1.0)
*Falciparum*	A	4.3 (1.0–7.6)	3.1 (1.7–4.6)	1.8 (1.4–2.3)	3.0 (2.3–3.7)	2.0 (1.5–2.5)	3.9 (3.0–4.9)	0.6 (0.5–0.8)	0.8 (0.6–1.0)
	I	0.6 (0.4–0.7)	0.7 (0.5–08)	2.7 (2.0–3.4)	5.8 (4.4–7.1)	2.2 (1.2–3.1)	5.0 (4.2–5.9)	0.4 (0.3–0.5)	0.6 (0.5–0.8)
	S	1.5 (1.0–2.0)	1.0 (0.8–1.2)	2.2 (1.8–2.6)	4.6 (4.0–5.2)	2.0 (1.6–2.6)	4.7 (4.0–5.3)	0.5 (0.4–0.6)	0.7 (0.6–0.8)
Total		4.6 (3.2–5.9)	1.5 (1.2–1.8)	1.7 (1.6–1.8)	3.3 (3.0–3.6)	1.5 (1.3–1.7)	3.3 (3.0–3.6)	1.1 (0.9–1.3)	0.9 (0.8–0.9)

*A* autochthonous, *I* imported, *S* sum

^a^All of the ratios examined statistically using multilevel Poisson regression with interaction terms for the phase (CP in compare to the EP). In all of the models, the interaction terms between the phase and the ratios were statistically significant except for the Iranian to non-Iranian ratio in imported vivax cases

^b^Given the high coverage of malaria laboratories and their accessibility, Passive Case Detection (PCD) takes place in all endemic rural and urban areas by examining blood smears for all suspected cases who attend the health units. Active Case Detection (ACD) is carried out as a routine activity by health worker through household visits in all endemic rural areas but in the urban areas it is limited to the active foci

## Discussion

The present study shows that the decline of malaria trends across malaria-endemic areas of Iran has significantly accelerated since the beginning of the malaria elimination programme. In the years subsequent to commencing the elimination programme (EP), autochthonous cases have declined faster (compared to the CP).

Since the elimination programme had commenced 5 years earlier, using suitable interventions, it was expected that the declining trend of malaria cases, which had already started around 10 years earlier, be accelerated. The findings of the present study have confirmed this.

Comparisons have been made between the two phases across different categories. Although the decline in all the studied categories was accelerated, the results were to some extent different for each category. The greatest difference in the annual decline across the two periods was observed in the autochthonous malaria cases, where the annual incidence showed an average of 35 % per year in the EP, indicating a 28 % greater decline compared to the CP. Higher influence of the interventions on reducing local transmission cases (as compared to imported cases) was consistent with other studies across different areas of the world, currently in the elimination phase or planning for commencing it [10, 11]. In other words, the interventions have managed successfully to decrease the transmission of disease across the malaria-endemic areas.

The lowest difference in the annual incidence decline, during the two periods, concerned falciparum malaria (an approximately 7 % difference in the decline of incidence in the two periods). There are two reasons for the lower change in annual decline of falciparum malaria (Table 1): first, the declining trend in falciparum malaria cases had already intensified prior to the elimination programme and second, the share of imported falciparum cases has gradually increased during the elimination years, which was less affected by the planned interventions in EP. Higher sensitivity *of P. falciparum* parasite (compared to *P.vivax* parasite) to interventions, as well as, greater decline of local transmission cases (compared to imported cases), have been mentioned in different studies [11, 12].

Imported and non-Iranian malaria cases, similar to falciparum cases, were less affected by the elimination programme. The majority of the imported cases were non-Iranians. There is a large flow of illegal immigrants who are hidden populations, and are mostly temporary residents of these areas, and therefore, their identification and regular surveillance is difficult. The lower difference of annual decline of malaria incidence (between CP and EP), is probably a function of these phenomena.

The Iranian to non-Iranian ratio was decreased in the EP compared to CP. The greater share of imported cases (which were mostly non-Iranian) over autochthonous cases in the EP, may be considered as a reason for smaller Iranian/non-Iranian ratio, in the EP. Studies in regions with similar endemicity have reported an increase in the imported cases versus locally transmitted cases in malaria elimination phase [11, 13].

The present study observed a declining trend for both imported and non-Iranian cases in the EP, however, at a lower speed than that of local transmission (Table 2). Since the majority of non-Iranian population consist of illegal immigrants, there were no data available on the number these people in each county. Therefore, caution should be taken when interpreting the Iranian to non-Iranian ratios.

The ratio of cases over 15/under 15, in the EP versus CP was increased. Younger population is usually considered as resident population with low regional relocation. Malaria incidence in this category highlights the possibility of local transmission, whereas incidence in population over 15 intensifies the possibility of imported cases, of course, where strong epidemiological evidence is present. Other studies have also observed an increase of adult cases upon witnessing a decline in local transmission cases, especially in the elimination phase [13, 14]. The increase in the ratio may, somehow, indicate a reduction in local transmission cases in the EP and an increased share (not number) of imported cases.

Increased male/female ratio also confirms the declined share of local transmission, as women, compared to men, have lower relocations, and the possibility of local malaria transmission seems to be higher for them. In the EP, as local transmission declines, males are expected to have an increased share of the malaria incidence [13].

Imported and non-Iranian cases were less benefited from the elimination programme. It would be prudent to improve malaria surveillance system for monitoring illegal population movements in the endemic areas and also for rapid and effective case detection and treatment. The mapping of malaria incidence showed that the eastern counties that are located near the border areas are at high risk for malaria transmission. Therefore, the next malaria elimination should put more emphasis on intensified activities in these regions.

### Limitations of the study

Notwithstanding the fact that the role of variables such as climate and socioeconomic conditions has been proved in changes of malaria incidence, they were considered constant in this study, and the effects of the interventions of the elimination programme was instead examined. Certainly, taking into account these variables along with the impact of the elimination programme will provide us with a better interpretation of the respective changes. On the other hand, defining each and every intervention as an independent variable enables the examination of manner and degree of influence of these variables, separately. Moreover, considering the trends in the incidence of malaria in the neighbouring countries can lead to an enhanced assessment of the observed patterns in Iran.

## Conclusions

Subsequent to the commencement of the elimination programme, the decline of malaria cases has been significantly accelerated in malaria-endemic areas in Iran. Local transmission cases have undergone the highest decline as a result of the elimination programme. The epidemiological pattern of malaria in the endemic areas confirms decreased transmission and a move towards the elimination of this disease.

## Abbreviations

CP: control and pre-elimination phase
EP: elimination phase

## Footnotes

1 It must be noted that the current population and area of active foci of malaria is much less than the area of the study, due to gradual narrowing of these foci over the past few years, owing to the administered interventions.

2 Local transmission.

3 Incidence rate ratio.

## References

[1] WHO. MDG 6: combat HIV/AIDS, malaria and other diseases. Geneva: World Health Organization; 2015. http://www.who.int/topics/millennium_development_goals/diseases/en/. Accessed 5 July 2015.

[2] WHO (2015). World malaria report 2015.

[3] Edrissian G (2006). Malaria in Iran: past and present situation. Iran J Parasitol.

[4] Mohammadi M, Ansari-Moghaddam A, Raiesi A, Rakhshani F, Nikpour F, Haghdost A (2011). Baseline results of the first malaria indicator survey in Iran at household level. Malar J.

[5] Raiesi A, Nikpour F, Ansari-Moghaddam A, Ranjbar M, Rakhshani F, Mohammadi M (2011). Baseline results of the first malaria indicator survey in Iran at the health facility level. Malar J.

[6] Raeisi A, Gouya MM, Nadim A, Ranjbar M, Hasanzehi A, Fallahnezhad M (2013). Determination of malaria epidemiological status in Iran’s malarious areas as baseline information for implementation of malaria elimination program in Iran. Iran J Public Health.

[7] Karunaweera ND, Galappaththy GN, Wirth DF (2014). On the road to eliminate malaria in Sri Lanka: lessons from history, challenges, gaps in knowledge and research needs. Malar J.

[8] Maude RJ, Nguon C, Ly P, Bunkea T, Ngor P, de la Torre SEC (2014). Spatial and temporal epidemiology of clinical malaria in Cambodia 2004–2013. Malar J.

[9] Fekri S, Vatandoost H, Daryanavard A, Shahi M, Safari R, Raeisi A (2014). Malaria situation in an endemic area, southeastern iran. J Arthropod-borne Dis.

[10] Yin JH, Yang MN, Zhou SS, Wang Y, Feng J, Xia ZG (2013). Changing malaria transmission and implications in China towards National Malaria Elimination Programme between 2010 and 2012. PLoS One.

[11] Coleman M, Al-Zahrani MH, Hemingway J, Omar A, Stanton MC, Thomsen EK (2014). A country on the verge of malaria elimination—the Kingdom of Saudi Arabia. PLoS One.

[12] Bousema T, Drakeley C (2011). Epidemiology and infectivity of *Plasmodium falciparum* and *Plasmodium vivax* gametocytes in relation to malaria control and elimination. Clin Microbiol Rev.

[13] Cotter C, Sturrock HJ, Hsiang MS, Liu J, Phillips AA, Hwang J (2013). The changing epidemiology of malaria elimination: new strategies for new challenges. Lancet.

[14] Griffin J, Ferguson N, Ghani A (2014). Estimates of the changing age-burden of Plasmodium falciparum malaria disease in sub-Saharan Africa. Nat Commun.

